# Helium-neon laser pre-treatment optimizes nutrient use efficiency and yield in garlic plant

**DOI:** 10.3389/fpls.2026.1821225

**Published:** 2026-05-21

**Authors:** Eltohamy A. A. Yousef, Abdelghafar M. Abu-Elsaoud

**Affiliations:** 1 Department of Horticulture, Faculty of Agriculture, Suez Canal University, Ismailia, Egypt; 2 Department of Biology, College of Science, Imam Mohammad Ibn Saud Islamic University (IMSIU), Riyadh, Saudi Arabia

**Keywords:** electromagnetic radiation, garlic, nitrogen, nutrient utilization efficiency, phosphorus, potassium

## Abstract

Garlic (*Allium sativum* L.) exhibits limited agronomic traits and nutrient use efficiency under conventional cultivation, necessitating innovative non-chemical approaches to enhance productivity. This study evaluated helium-neon (He-Ne) laser and ultraviolet (UV_A+B_) radiation as sustainable pre-treatment strategies to optimize garlic growth, yield, and nutrient utilization. Garlic cloves were pre-treated with control (no irradiation), He-Ne laser (1, 5, 30, or 60 minutes), or UV_A+B_ ra6diation (1, 5, 30, or 60 minutes). Results demonstrated that He-Ne laser produced dose-dependent improvements, with 60-minute exposure maximizing plant height (20.5%), shoot fresh weight (48.1%), bulb fresh weight (29.1%), bulb yield (29.0%), and nutrient use efficiency (nitrogen: 146.6%, phosphorus: 85.1%, potassium: 78.4%). In contrast, UVA+B radiation exhibited a biphasic response, with moderate benefits at low doses (1–5 minutes) but progressive decline at extended exposure (30–60 minutes), reducing yield by 10.7% and nutrient use efficiency by 14.9–29.9%. Response Surface Methodology confirmed 60-minute He-Ne laser pre-treatment as the optimal dose, representing a sustainable strategy for enhancing garlic productivity. These findings demonstrate the potential of laser-based technologies for sustainable vegetable production and warrant further investigation into cultivar-specific optimization and field-scale applications for global food security.

## 1 Introduction

Garlic (*Allium sativum* L.) is a major horticultural crop valued for its culinary, medicinal, and economic importance. As it reproduces asexually, improving productivity and nutritional quality remains a key goal. Given the emphasis on sustainable fertilization and nutrient use efficiency, electromagnetic (EM) radiation treatments offer promising non-chemical photostimulation strategies to enhance vital physiological processes, including photosynthesis, ion transport, and nutrient use efficiency (Abu-Elsaoud, 2013; Abu-Elsaoud and Tuleukhanov, 2013; Okla et al., 2021). These physical approaches provide energy-efficient alternatives to chemical inputs required for plant growth and development (Jacobo-Velázquez et al., 2022; Dudareva, 2024; Liaqat et al., 2024). Among EM tools, helium-neon (He-Ne) laser and ultraviolet (UV) radiation represent as promising biostimulants, eliciting physiological and biochemical responses that improve major horticultural traits such as productivity and quality (Aladjadjiyan, 2007; Abu-Elsaoud and Abdel-Azeem, 2019; Metwally et al., 2020; Awaad, 2021).

It was reported that the pre-sowing He-Ne laser treatment effectively accelerated growth and yield-related traits in several crop species, such as coriander and fennel (Osman et al., 2009), sunflower (Mahmood et al., 2021), ashwagandha (Thorat et al., 2021),wheat (Aslam et al., 2022), soybean (Klimek-Kopyra et al., 2022), clover (El-Raouf Ahmed et al., 2023), gladiolus (Hassan et al., 2024), and sugar beet (Rudikovskii et al., 2025). Also, He-Ne lasers treatment enhanced tolerance against drought (Qiu et al., 2017) and salinity (Thorat et al., 2024), mitigated heavy metal toxicity (Różanowski et al., 2020; Zhu et al., 2022; Amooaghaie and Banisharif, 2025). In addition, He-Ne laser pre-treatment demonstrated consistent efficiency in enhancing seed germination and seedling vigor across diverse crop species, including brinjal (Muthusamy et al., 2012), triticale (Możdżeń et al., 2020), safflower (Perveen et al., 2021), and wheat (Jamil et al., 2013), and significantly enhanced biomass accumulation, mineral nutrient uptake, and essential oil production (Okla et al., 2021), which in turns directly enhanced the plant productivity and nutritional quality of harvested crops.

He-Ne laser emits coherent, monochromatic red light at 632.8 nm wavelength with photon energy of 1.96 eV. This wavelength allows deep tissue penetration, enabling phytochrome-mediated signaling. It produces highly concentrated light energy without significant thermal effects, allowing precise cellular targeting. The physiological mechanisms underlying He-Ne laser biostimulation are well-characterized. For instance, He-Ne laser enhances seed germination, plant growth, yield, and nutrient content through distinct biological pathways. Specifically, it interacts with the phytochrome system and consequently modulate metabolic pathway and endogenous phytohormone balance (Metwally et al., 2020; Swathy et al., 2023; Dudareva, 2024), which directly enhance photosynthesis, carbon allocation and synthesis of metabolic compounds (Okla et al., 2021; Swathy et al., 2021). In addition, He-Ne laser pre-treatment protects plants from oxidative injury by improving pigment and metabolite integrity through enhanced antioxidant enzyme activities (Aslam et al., 2022; Banisharif and Amooaghaie, 2025), thereby maintaining photosynthetic efficiency and nutrient availability. Additionally, He-Ne treatment stimulates mitotic activity in root-tip meristem cells and upregulate transcripts related to photosynthesis, nutrient uptake and transport, and stress response (Shafique et al., 2017; Qiu et al., 2017; Różanowski et al., 2020; Zhu et al., 2022). These combined physiological mechanisms result in enhanced biomass assimilation, growth, yield, and improved biochemical and nutraceutical values across diverse plant species.

UV radiation encompasses wavelengths between 100 and 400 nm, located beyond the visible spectrum. UV radiation is subdivided into three categories: UVA (315–400 nm), UVB (280–315 nm), and UVC (100–280 nm). UV radiation is now recognized as a valuable tool for physiological modulation of horticultural traits (Liu et al., 2013; Correa et al., 2023; Mmbando, 2024; Escobar-Hernández et al., 2025). Practically, UV supplementation increased biomass and antioxidant content in leafy greens and induced water stress tolerance in tomato and bell pepper (Kang et al., 2018; Chen et al., 2019; Lee et al., 2019; Rodríguez-Calzada et al., 2019; Yan et al., 2021; He et al., 2021; Kang et al., 2022; Escobar-Hernández et al., 2024). However, UV radiation might negatively affect photosystems, chlorophyll content, photosynthetic efficiency, mineral content in plant tissues (Teramura, 1983; Zhang and Jiang, 2019). The physiological basis for these contrasting effects might lie in the dose-dependent activation of defense mechanisms. Whereas low-dose UV exposure induces eustress, which stimulates innate defense pathways that enhance photosystem function, carboxylation efficiency, and accumulation of protective secondary metabolites, thereby improving biomass production, nutritional quality, and postharvest shelf-life (Zhang and Jiang, 2019; Darré et al., 2022; Viršilė et al., 2023; Li et al., 2024). Conversely, extended UV exposure might trigger distress responses that predominate overprotective mechanisms, resulting in photosystem damage, excessive oxidative stress, and impaired nutrient acquisition efficiency, which collectively reduce growth and yield performance (Teramura, 1983; Zhang and Jiang, 2019).

Both He-Ne laser and UV radiation highly exhibit dose-dependent and species-dependent effects, where an optimal dose can significantly enhance growth and nutrient content, whereas excessively long exposure may have inhibitory effects (Możdżeń et al., 2020). The efficiency of He-Ne laser and UV pre-treatment is influenced by multiple factors, such as irradiation dose, frequency, seed material characteristics, and species-specific photoblastism (light-dependent germination). Practically, He-Ne laser biostimulation demonstrated a clear dose-response relationship, with optimal doses varying by species: 2 minutes for sunflower and wheat (Mahmood et al., 2021; Aslam et al., 2022), 5 minutes for fennel and clover, 20 minutes for coriander (Osman et al., 2009; El-Raouf Ahmed et al., 2023). While suboptimal or excessive doses produce low effectiveness or inhibitory effects (Amooaghaie and Banisharif, 2025; Banisharif and Amooaghaie, 2025). Similarly, UV radiation effects are dose-dependent and species-specific, with high UV-B doses reduced growth parameters, chlorophyll content, photosynthetic parameters, and nutrient concentrations (Teramura et al., 1991; Zuk-Golaszewska et al., 2003). While low-medium doses of UV increased fruit set, growth, and the metabolite profile as well as tuber quality (Darras et al., 2020; Qi et al., 2020; Fitzner et al., 2023; Skowron et al., 2025). These findings demonstrate that both radiation types produce dose-dependent and species-dependent responses, which necessitates species-specific optimization for agricultural applications to achieve high horticultural outcomes.

Although the individual biostimulatory effects of He-Ne laser and UV radiation are well-characterized, comparative studies within a single crop remain absent from scientific literature. Furthermore, garlic has not been systematically evaluated for its physiological and horticultural responses to either He-Ne laser or UV radiation, despite its importance as a horticultural crop. Therefore, the overall objective of this research was to investigate whether He-Ne laser irradiation and/or UV_A+B_ radiation differentially modulates mineral content, nutrient uptake and use efficiency in garlic. To test this hypothesis, our specific objectives were to: (1) evaluate the dose-dependent effects of He-Ne laser (1, 5, 30, 60 minutes) and UV_A+B_ radiation (1, 5, 30, 60 minutes) on garlic growth, yield, and mineral content. (2) compare the physiological mechanisms underlying He-Ne laser and UV_A+B_ radiation effects on nutrient uptake and nutrient use efficiency. (3) Identify optimal application parameters for each radiation type to maximize garlic productivity and nutritional quality.

## 2 Materials and methods

### 2.1 Plant material and experimental design

The plant material used in this study was uniform and healthy cloves of garlic cv. Seds-40, a cultivar widely grown throughout Egypt.

### 2.2 Irradiation treatments

To ensure uniform hydration, selected cloves were soaked in running tap water for 12 hours prior to treatment. Later, He-Ne laser (632.8 nm, 15 mW, ~10 mW/mm²) and UV_A+B_ radiation (λ= 280–400 nm, λmax=340 nm, 20 W) were applied to moistened garlic cloves at a distance of 250 mm and 450 mm, respectively. Irradiation doses of 0 (control), 1, 5, 30, and 60 minutes were selected based on preliminary dose-response experiments and literature recommendations. Treatment groups included: (1) non-irradiated control group, (2) He-Ne laser treated cloves with radiation duration of 1, 5, 30, and 60 minutes, and (3) ultraviolet (UV_A+B_) treated cloves with radiation duration of 1, 5, 30, and 60 minutes. All detailed information about radiation equipment specifications and irradiation parameters for He-Ne laser and ultraviolet (UVA+B) treatments is provided in **Table 1**.

**Table 1:** Radiation equipment specifications and irradiation parameters for He-Ne laser and UV_A+B_ treatments.

Parameters	He-Ne laser	UV_A+B_
Wavelength	632.8 nm	300–400 nm
Power	15 mW	20 W
Beam size	1.5 mm	600 x 45 mm
Lamb Model	Spectra-Physics 124A He-Ne Laser	Turbo Black Light Blue FL20T8/BLB
Lamp dimensions/Specs	Beam diameter: 1.1 mm Polarization: Linear Watts: 15 mW	Diameter: 38.10 mm Length: 609.60 mm Watts: 20 W
λmax	632.8	340 nm
Distance from sample	250 mm	450 mm
Wave emission	Continuous (CW)	Continuous (CW)
Irradiation times	Single	Single
Type of irradiation	One shot pre-sowing irradiation	One shot pre-sowing irradiation
Duration of treatment (minutes)	0, 1, 5, 30 and 60	0, 1, 5, 30 and 60
Temperature of	25 C°	25 C°

### 2.3 Experimental design and agricultural practices

The field experiment was performed out at the Research Experimental Farm of the Faculty of Agriculture, Suez Canal University, located in Ismailia, Egypt. The soil at the experimental site was sandy loam with the following physicochemical properties: 92% sand, 6% clay, and 2% loam; pH 7.94; electrical conductivity (EC) 0.56 dS m^−^¹; available nitrogen (N) 20 ppm; available phosphorus (P) 25 ppm; and available potassium (K) 40 ppm.

Prior to planting, the experimental field was cleared and ploughed to a depth of 25 cm. During final soil preparation, organic matter and mineral amendments were added: 20 m³ of well-decomposed cow manure, 323 kg of calcium superphosphate (15.5% P_2_O_5_), and 60 kg of sulfur per feddan. These amendments were thoroughly mixed into the soil to ensure uniform distribution.

The field trial arranged in a randomized complete block design (RCBD) with three replicates. Each experimental plot consisted of 50 plants. Control and irradiation-treated garlic cloves were sown on 10^th^ October 2023 under a drip irrigation system, arranged in two rows (one row on each side of the drip hose) at 10 cm intra-row spacing and 100 cm inter-row spacing between drip hoses. Nutrient supplementation was provided through a fertigation system: 358 kg/feddan of ammonium nitrate (33.5% N) and 156 kg/feddan of potassium sulfate (48% K_2_O). All other agronomic practices, including irrigation timing, integrated pest and disease management, as well as weed control, were implemented in accordance with the official recommendations of the Egyptian Ministry of Agriculture and Land Reclamation.

### 2.4 Measurements

At maturity stage (180 DAS), five randomly selected plants per plot were harvested and carefully separated into three distinct plant parts: shoot, bulb, and root. Measured parameters included: plant height (cm), number of leaves per plant, shoot fresh weight (g), bulb fresh weight, and roots fresh weight (g), bulb diameter (cm), dry matter content (%) of shoot, bulb, and root, and soluble solids content (SSC, %). Additionally, two composite indices were calculated: the bulbing ratio (Mann, 1952), and the total bulb yield by harvesting all remaining plants in each plot, weighing the fresh bulbs, and expressing the final yield in kilograms per feddan (kg fed^−^¹). Total leaf chlorophyll content was measured at fifth leaf at 100 days after sowing (DAS) using a SPAD-502Plus portable chlorophyll meter (SPAD value).

### 2.5 Determination of N, P and K content

The plant tissue, leaves, bulbs, and roots, of five representative plants from each replicate were oven-dried at 70 °C to constant weight, then individually ground into a fine, homogeneous powder using a mortar and pestle. For nutrient analysis, a 0.5 g subsample of each powdered tissue was subjected to wet acid digestion using a mixture of sulfuric acid (H_2_SO_4_) and hydrogen peroxide (H_2_O_2_) to oxidize organic matter. The resulting digestate was quantitatively transferred to a volumetric flask and brought to a final volume of 100 mL using sterilized distilled water. Later macronutrient concentrations were subsequently countified in shoot, bulb and root samples using standardized analytical methods. Total N content (mg g^−^¹) was quantified according to the modified micro-Kjeldahl procedure (Jackson, 1973). Total P content (mg g^−^¹) concentration was determined via colorimetric analysis (Black, 1965). Total K content (mg g^−^¹) concentration was measured using flame photometry (Jackson, 1973). All analyses were performed in triplicate to ensure accuracy and reproducibility.

### 2.6 Nutrient uptake, use and productive efficiency.

To evaluate N, P, and K utilization efficiency in response to irradiation treatments, three complementary indices were calculated (White et al., 2021; Ma et al., 2022):

Nutrient Uptake Efficiency (NUE, %) was calculated as the ratio of total plant nutrient accumulation to total nutrient input:


$$\text{NUE}=\frac{\text{Total plant nutrient uptake }({\text{kg fed}}^{-1})}{\text{Total nutrient input }({\text{kg fed}}^{-1})}\times 100$$

Nutrient Use Efficiency (NUtE, %) was determined as the ratio of nutrient accumulation in the economically important bulb tissue to total nutrient input:


$$\text{NUtE}=\frac{\text{Bulb nutrient concentration }({\text{kg fed}}^{-1})}{\text{Total nutrient input }({\text{kg fed}}^{-1})}\times 100$$

Nutrient Productive Efficiency (NPE, kg/kg) was calculated as the ratio of bulb fresh biomass production to total nutrient input:


$$\text{NPE}=\frac{\text{Bulb fresh weight }({\text{kg fed}}^{-1})}{\text{Total nutrient input }({\text{kg fed}}^{-1})}$$

Where total nutrient input represents the cumulative nutrient pool comprising indigenous soil nutrient content, nutrient supplied through organic amendments, and nutrient supplied through mineral fertilizers. Total plant nutrient uptake represents the cumulative nutrient concentration across all plant organs (shoot, bulb, and root). Bulb nutrient concentration specifically quantifies nutrient accumulation in bulb tissue only.

### 2.7 Statistical analysis

All collected data were subjected to statistical analysis using R software version 4.4.0 (R Core Team, 2024). A two-way analysis of variance (ANOVA) was performed using the *aov* function from the stats package to determine the significance of the main effects and their interaction. Following the ANOVA, mean comparison was conducted using Tukey’s Honestly Significant Difference (HSD) multiple range test at a significance level of *p* ≤ 0.01. This *post-hoc* analysis was implemented using the *HSD.test* function from the *agricolae* package (de Mendiburu, 2023). All results are presented as mean ± standard deviation (SD) calculated from 15 plants (5 plants per replicate plot) in a randomized complete block design with three replicates (n = 15).

The principal component analysis (PCA) was performed to evaluate the relationship between variables of different traits and the possible impact of treatment with the help of R code “FactoMineR” and “ggplot2” packages.

To identify the optimal pre-sowing radiation conditions for maximizing garlic bulb yield, a Response Surface Methodology (RSM) approach was applied using a second-order polynomial regression model (Iqbal et al., 2013). The model predicted bulb yield (kg fed^−^¹) as a function of radiation type and dose duration. Radiation type was encoded using two binary dummy variables, with the non-irradiated control as the reference level. Since dummy variables are binary, their squared terms were not independently estimable, and the interaction between the two radiation dummies is structurally zero due to mutual exclusivity. The estimable form of the fitted model:


$$Y\;=\;\beta_{0}\;+\;\beta_{1}X_{1}\;+\;\beta_{2}X_{2}\;+\;\beta_{3}X_{3}\;+\;\beta_{12}(X^{1}X^{2})\;+\;\beta_{13}X_{1}X_{3}\;+\;\beta_{11}X_{1}²\;+\;\epsilon$$

where *Y* is the predicted bulb yield, *X*_1_ is dose duration (min), *X*_2_ and *X*_3_ are dummy variables for He-Ne Laser and UVA+B treatments, respectively, and ϵ is the error term. Model significance was evaluated at *p* ≤ 0.05 using the rsm package.

## 3 Results

### 3.1 Effect of He-Ne laser and UV _A+B_ on growth and yield of garlic

The two-way ANOVA demonstrated that radiation type was a primary driver of phenotypic variation and significantly affected all measured growth and yield traits except leaf number. In contrast, radiation dose significantly affected only shoot dry matter and chlorophyll content. The radiation × dose interaction was significant for all traits except leaf number and SSC (**Table 2**).

**Table 2:** Growth and yield traits of garlic in response to various doses of He-Ne laser and UV_A+B_ radiation.

Radiation	Dose (min.)	Plant height (cm)	Number of leaves	Shoot fresh weight (g)	Bulb fresh weight (g)	Root fresh weight (g)	Shoot dry matter (%)	Bulb dry matter (%)	Root dry matter (%)	Bulb diameter (cm)	Bulbing ratio	Soluble solid content (SSC, %)	Chlorophyll content (SPAD value)	Total bulb yield (Kg/feddan)
Control	0	50.18 ± 9.03b	7.70 ± 0.95a	82.21 ± 8.83c	50.02 ± 6.27bc	2.30 ± 0.48bc	13.70 ± 1.06bcd	31.96 ± 2.85d	37.90 ± 3.54a	5.47 ± 0.63bc	0.19 ± 0.01d	38.90 ± 2.6a	65.20 ± 12.15b	8281.88 ± 459.83cde
UV_A+B_	1	55.81 ± 7.76ab	8.00 ± 1.05a	105.24 ± 7.79ac	52.19 ± 6.26bc	2.80 ± 0.79bc	14.90 ± 0.74ab	34.18 ± 3.11cd	39.00 ± 2.91a	5.77 ± 0.43abc	0.23 ± 0.02abcd	33.20 ± 3.97b	66.20 ± 8.69b	8811.25 ± 1165.89bcd
	5	56.94 ± 6ab	8.40 ± 0.7a	102.27 ± 9.9b	56.80 ± 9.42ab	2.60 ± 0.70bc	14.30 ± 1.16abc	32.11 ± 1.87d	39.00 ± 2.16a	5.76 ± 0.41abc	0.25 ± 0.03ab	34.20 ± 4.71b	69.70 ± 9.21b	8885.88 ± 736.23bc
	30	49.88 ± 4.09b	8.20 ± 0.42a	96.18 ± 8.61bc	48.56 ± 5.24bc	2.30 ± 0.48bc	12.30 ± 1.06de	31.59 ± 3.91d	33.10 ± 2.42b	5.13 ± 0.83c	0.21 ± 0.03bcd	34.10 ± 3.78b	68.60 ± 7.04b	7657.50 ± 771.08de
	60	50.32 ± 4.47b	8.10 ± 0.74a	93.48 ± 15.38bc	46.40 ± 3.07c	2.00 ± 00c	11.00 ± 0.94e	31.78 ± 3.88d	33.00 ± 1.94b	5.10 ± 0.77c	0.20 ± 0.02cd	32.70 ± 2.16b	65.50 ± 7.96b	7397.00 ± 773.34e
	**Mean**	**53.24 ± 6.4**	**8.18 ± 0.75**	**99.29 ± 11.44**	**50.99 ± 7.35**	**2.43 ± 0.64**	**13.13 ± 1.84**	**32.42 ± 3.34**	**36.03 ± 3.79**	**5.44 ± 0.7**	**0.22 ± 0.03**	**33.55 ± 3.68**	**67.5 ± 8.13**	**8187.91 ± 1082.15**
He-Ne laser	1	56.94 ± 4.93ab	8.00 ± 0.82a	102.04 ± 12.67b	55.76 ± 5.88ab	3.00 ± 0.67ab	13.40 ± 1.26cd	35.62 ± 2.91bcd	36.90 ± 2.13ab	5.98 ± 0.65abc	0.23 ± 0.06abcd	37.00 ± 2.87ab	71.60 ± 6.82b	9519.63 ± 756.69ab
	5	56.68 ± 8.27ab	8.60 ± 0.84a	102.09 ± 14.39b	55.93 ± 6.18ab	3.00 ± 0.67ab	14.80 ± 0.79abc	36.79 ± 3.63abc	37.60 ± 2.46a	6.14 ± 0.65ab	0.24 ± 0.05abc	34.60 ± 2.46ab	69.40 ± 13.67b	9591.38 ± 951.39ab
	30	59.93 ± 5.14a	8.30 ± 0.67a	110.50 ± 14.44ab	57.46 ± 6.15ab	3.00 ± 0.47ab	15.00 ± 0.82ab	38.58 ± 2.6ab	39.60 ± 3.72a	6.35 ± 0.49ab	0.27 ± 0.04a	35.30 ± 1.83ab	70.40 ± 9.13b	9906.88 ± 1048.41ab
	60	60.45 ± 6.94a	8.50 ± 0.97a	121.77 ± 14.71a	64.60 ± 6.05a	3.80 ± 0.63a	15.50 ± 1.43a	40.52 ± 1.89a	40.60 ± 3.17a	6.56 ± 0.67a	0.27 ± 0.02a	35.10 ± 3.00ab	88.10 ± 7.50a	10682.88 ± 582.92a
	**Mean**	**58.5 ± 6.45**	**8.35 ± 0.83**	**109.1 ± 15.81**	**58.44 ± 6.88**	**3.20 ± 0.69**	**14.68 ± 1.33**	**37.88 ± 3.30**	**38.68 ± 3.20**	**6.26 ± 0.63**	**0.25 ± 0.05**	**35.5 ± 2.64**	**74.88 ± 12.10**	**9925.19 ± 943.99**
Analysis of variance (ANOVA)														
F_(Radiation)_		9.91***	2.57	20.89***	16.73***	20.90***	21.86***	36.99***	9.23***	18.51***	17.99***	12.72***	7.97***	47.51***
F _(Dose)_		0.36	1.27	0.77	1.16	0.81	5.84**	1.12	2.21	0.39	1.01	0.15	3.19*	1.16
F _(Radiation * Dose)_		3.67*	0.22	7.07***	8.62***	7.42***	30.75***	5.35**	16.83***	4.78**	7.41***	1.09	6.15***	11.53***

Data are presented as mean ± SD (n=15). Values represent measurements from 15 plants (5 per replicate plot) in a randomized complete block design with three replicates. SD reflects variation among all sampled plants. Means followed by the same letter are not significantly different according to Tukey’s multiple range test. Significance levels: *p < 0.05, **p < 0.01, ***p < 0.001.Bold values indicate the mean of each radiation type (He-Ne laser or UVA+B) across all dose levels.

Among the radiation treatments evaluated, He-Ne laser treatment resulted in significantly higher values for all measured growth traits compared to UV_A+B_ and control treatments. Specifically, He-Ne laser treatment produced the maximum values of plant height of 58.50 cm, number of leaves of 8.35, shoot fresh weight of 109.10 g, chlorophyll content of 74.88 SPAD values, shoot dry matter of 14.68%, root fresh weight of 3.20 g, and root dry matter of 38.68%. Correspondingly, He-Ne laser treatment significantly enhanced all yield and yield-related traits, achieving the maximum values of bulb fresh weight of 58.44 g, bulb dry matter of 37.88%, bulb diameter of 6.26 cm, bulbing ratio of 0.25, and total yield of 9925.19 kg/feddan, compared to control (8281.88 kg/feddan) and UV_A+B_ treatments (8187.91 kg/feddan).

In contrast, UV _A+B_ treatment resulted moderate increases in plant height (53.24 cm), number of leaves (8.18), shoot fresh weight (99.29 g), bulb fresh weight (50.99 g), root fresh weight (2.43 g), bulb dry matter (32.24%), bulbing ratio (0.22), and chlorophyll content (67.5 SPAD value) compared to control treatment. However, these increases were not statistically significant, except for shoot fresh weight. Moreover, UV_A+B_ treatment decreased shoot dry matter (13.13%), root dry matter (36.03%), bulb diameter (5.44 cm), SSC (33.55%), and total bulb yield (8187.91 kg/feddan) compared to control. Nevertheless, these reductions were not statistically significant except for SSC.

Data in **Table 2** clearly states that both He-Ne laser and UV_A+B_ exposure duration demonstrated a dose-dependent relationship with growth and yield parameters. When He-Ne laser exposure extended from 1 to 60 minutes, progressive increases occurred across most of measured traits. For instance, plant height increased by 6.2%, number of leaves by 6.3%, shoot fresh weight by 19.3%, root fresh weight by 26.7%, shoot dry matter by 15.7%, root dry matter by 10.0%, and chlorophyll content by 23.0%. Furthermore, bulb fresh weight increased by 15.9%, bulb dry matter by 13.8%, bulb diameter by 9.7%, bulbing ratio by 17.4%, and total yield by 12.2% when He-Ne laser exposure was extended from 1 to 60 minutes. However, SSC decreased by 5.1% with prolonged He-Ne laser exposure (**Table 2**).

Compared to control treatment, He-Ne laser at 60 minutes produced substantial improvements across all growth parameters: plant height increased by 20.5%, leaf number by 10.4%, shoot fresh weight by 48.1%, shoot dry matter by 13.1%, root fresh weight by 65.2%, root dry matter by 7.1%, and chlorophyll content by 35.1%. Yield-related traits also showed similarly positive enhancements: bulb fresh weight increased by 29.1%, bulb dry matter by 26.8%, bulb diameter by 19.9%, bulbing ratio by 42.1%, and total yield by 29.0%. Notably, SSC was decreased by 9.8% relative to control.

In contrast to He-Ne laser, UV_A+B_ radiation exhibited a fundamentally different dose-response pattern: maximum benefits occurred at short exposure durations (1 and 5 minutes), while extended exposures (30 and 60 minutes) produced progressive decline. Extending UV_A+B_ exposure from 1 to 60 minutes resulted in substantial reductions across most measured traits. Plant height declined by 9.8%, shoot fresh weight by 11.2%, root fresh weight by 28.6%, shoot dry matter by 26.2%, root dry matter by 15.4%, and chlorophyll content by 1.1%. Also, yield-related traits showed similar reduction: bulb fresh weight by 11.1%, bulb dry matter by 7.0%, bulb diameter by 11.6%, bulbing ratio by 11.8%, soluble solid content by 1.5%, and total yield by 16.1%.

At optimal doses (1 and 5 minutes), UV_A+B_ treatment enhanced most growth parameters compared to control: plant height increased by 11.2% and 13.5% respectively, shoot fresh weight by 28.0% and 24.4%, bulb fresh weight by 4.3% and 13.6%, and root fresh weight by 21.7% and 13.0%. Chlorophyll content increased by 1.5% and 6.9%, bulb diameter by 5.5% and 5.3%, bulbing ratio by 21.8% and 34.0%, and total yield by 6.4% and 7.3%. However, extended exposures (30 and 60 minutes) reversed these positive responses and reduced bulb fresh weight (2.9% and 7.2%), shoot dry matter (10.2% and 19.7%), bulb diameter (6.2% and 6.8%), and total yield (7.5% and 10.7%) compared to control treatment.

### 3.2 Effect of He-Ne laser and UV_A+B_ on nutrient content

Data in **Table 3** clearly revealed that radiation type significantly influenced content of all mineral elements (N, P, and K) across all plant tissues of garlic. While radiation dose had significant effects only on N and P content in shoot, N content in bulbs, and N and K content in root. The radiation × dose interaction effect was significant for all mineral elements across all plant tissues, except K content in shoot.

**Table 3:** Mineral content in shoot, bulb and root of garlic in response to various doses of He-Ne laser and UV_A+B_ radiation.

Radiation /dose (min.)		Plant tissue/NPK treatment (mg g^-1^)										
		Shoot			Bulb			Root		
		N	P	K	N	P	K	N	P	K
**Control**	--	18.85 ± 1.1cd	2.65 ± 0.22bc	21.15 ± 0.81d	14.40 ± 1.24de	2.06 ± 0.21bc	15.20 ± 1.23abcd	10.45 ± 0.96cd	1.53 ± 0.2abc	10.91 ± 0.84abc
**UV_A+B_**	1	20.79 ± 1.62bc	2.89 ± 0.41abc	24.14 ± 1.06bc	16.30 ± 1.49cd	2.09 ± 0.21abc	15.70 ± 1.49ab	11.69 ± 0.71bc	1.61 ± 0.15abc	10.63 ± 0.9bc
	5	22.37 ± 1.36ab	3.00 ± 0.23ab	25.37 ± 0.79abc	17.30 ± 1.06bc	2.14 ± 0.22abc	14.10 ± 1.2bcd	11.20 ± 1.23cd	1.45 ± 0.26bc	10.58 ± 0.98bc
	30	16.81 ± 2.57de	2.49 ± 0.23c	24.89 ± 1.22abc	14.85 ± 1.45de	1.89 ± 0.25cd	13.60 ± 1.26cd	10.75 ± 0.86cd	1.49 ± 0.25abc	10.06 ± 0.87c
	60	15.96 ± 1.68e	2.49 ± 0.19c	23.92 ± 1.97c	13.60 ± 1.07e	1.60 ± 0.24d	13.90 ± 0.99d	9.25 ± 0.87d	1.40 ± 0.16c	7.89 ± 0.7d
	**Mean**	**18.98 ± 3.25**	**2.72 ± 0.36**	**24.58 ± 1.41**	**15.51 ± 1.89**	**1.93 ± 0.31**	**14.33 ± 1.46**	**10.72 ± 1.29**	**1.49 ± 0.22**	**9.79 ± 1.41**
**He-Ne laser**	1	21.36 ± 0.98b	3.08 ± 0.25a	25.78 ± 0.79abc	15.70 ± 1.77cd	2.17 ± 0.18bcd	14.73 ± 1.09bcd	12.19 ± 1.11bc	1.59 ± 0.12abc	12.22 ± 1.52a
	5	22.50 ± 1.06ab	3.14 ± 0.13a	26.04 ± 0.74a	19.00 ± 1.25bc	2.08 ± 0.36abc	15.00 ± 1.05abcd	13.20 ± 1.14bc	1.54 ± 0.22abc	11.70 ± 1.42ab
	30	22.83 ± 1.4ab	3.11 ± 0.32a	26.36 ± 1.48a	21.80 ± 1.32a	2.48 ± 0.36a	15.60 ± 1.07abc	16.10 ± 2.13a	1.76 ± 0.32ab	11.10 ± 0.88abc
	60	23.80 ± 1.96a	3.01 ± 0.43ab	26.02 ± 2.13ab	21.60 ± 2.22a	2.33 ± 0.42ab	16.56 ± 1.38a	16.20 ± 1.4a	1.79 ± 0.21a	11.90 ± 0.74ab
	**Mean**	**22.62 ± 1.61**	**3.09 ± 0.3**	**26.05 ± 1.37**	**19.53 ± 2.98**	**2.27 ± 0.37**	**15.47 ± 1.32**	**14.42 ± 2.3**	**1.67 ± 0.24**	**11.73 ± 1.21**
Analysis of variance (ANOVA)										
F_(Radiations)_		58.44***	20.11***	56.46***	94.19***	13.85***	9.31***	105.10***	7.23**	36.31***
F_(Dose)_		11.88***	5.46**	1.89	10.34***	2.16	1.92	5.75**	1.38	8.77***
F_(Radiation* Dose)_		29.36***	3.50*	1.02	39.32***	9.03***	8.64***	29.49***	3.52*	9.45***

Data are presented as mean ± SD (n=15). Values represent measurements from 15 plants (5 per replicate plot) in a randomized complete block design with three replicates. SD reflects variation among all sampled plants. Means followed by the same letter are not significantly different according to Tukey’s multiple range test. Significance levels: *p < 0.05, **p < 0.01, ***p < 0.001.Bold values indicate the mean of each radiation type (He-Ne laser or UVA+B) across all dose levels.

Data in **Table 3** also shows that He-Ne laser treatments resulted in significantly higher mineral content (N, P, and K) across all plant tissues compared to UV_A+B_ and control treatments. He-Ne laser treatment produced the highest N, P and K content in shoot (22.62, 3.09, and 26.05 mg g^−^¹, respectively), bulb (19.53, 2.27, and 15.47 mg g^−^¹, respectively), and root (14.42, 1.67, and 11.73 mg g^−^¹, respectively). Also, UV_A+B_ treatment resulted in insignificant increases in N, P, and K content in shoot (18.98, 2.72, and 24.58 mg g^−^¹, respectively) and N content in bulb and root (15.51 and 10.72 mg g^−^¹, respectively) compared to control. On the other hand, UV_A+B_ treatment resulted in non-significant decreased in content of P and K in bulb (1.93 and 14.33 mg g^−^¹, respectively) and root (1.49 and 9.79 mg g^−^¹, respectively) compared to untreated plants.

Regarding the dose-dependent effects, He-Ne laser exposure duration demonstrated a clear dose-dependent relationship with mineral content in plant tissues. Extending He-Ne laser exposure from 1 to 60 minutes resulted in progressive increases in most mineral elements. N and K content increased in shoot by 11.4% and 0.9%, respectively. Similarly, N, P, and K content exhibited increases of 37.6%, 7.4%, and 12.4%, respectively, in bulb. Additionally, N and P content in root increased by 32.9% and 12.6%, respectively. However, content of P in shoot and K in root decreased by 2.3 and 2.6%, respectively, when He-Ne laser exposure was extended to 60 minutes. Compared to control, He-Ne laser treatment at 60 minutes exposure resulted in substantial increases in mineral content across all plant tissues. Specifically, N, P, and K content increased in shoot by 26.3, 13.6, and 23.0%, respectively, and in bulb by 50.0, 13.1, and 8.9%, respectively, as well as in root by 55.0, 17.0, and 9.1%, respectively.

In contrast to He-Ne laser effects, UV_A+B_ radiation demonstrated diminished mineral accumulation at extended exposure durations. Extending UV_A+B_ exposure from 1 to 60 minutes resulted in significant reductions in mineral content across all plant tissues. N, P, and K content in shoot decreased by 23.2, 13.8, and 0.9%, respectively, and decreased by 16.6, 23.4, and 11.5%, respectively, in bulb. Moreover, N, P, and K content in root showed a pronounced decline of 20.9, 13.0, and 25.8% when UV_A+B_ exposure was extended to 60 minutes.

Data presented in **Table 3** shows that UV_A+B_ treatment demonstrated a dose-dependent relationship with NPK accumulation in shoot, bulb, and root tissues. Compared to control plants, 5-minute UV_A+B_ exposure produced the maximum values for shoot N, P, and K (22.37, 3.00, and 25.37 mg g^−^¹, respectively), representing increases of 18.7, 13.2, and 20.0%, respectively. Similarly, bulb N increased from 14.4 in control to 17.3 mg g^−^¹ at 5-minute exposure (20.1% increase), and bulb P increased from 2.06 to 2.14 mg g^−^¹ (3.9% increase). Furthermore, root N increased from 10.45 in control to 11.69 mg g^−^¹ at 1-minute exposure (11.9% increase), and root P increased from 1.53 to 1.61 mg g^−^¹ at 1-minute exposure (5.2% increase). However, extending UV_A+B_ exposure beyond 5 minutes resulted in progressive decreases in nutrient accumulation across all tissues. At 30-minute exposure, N content decreased to 16.81, 14.85, and 10.75 mg g^−^¹ in shoot, bulb, and root, respectively. Also, further reductions were observed in N content to 15.96, 13.6, and 9.25 mg g^−^¹ in shoot, bulb, and root, respectively, at 60-minute exposure. Additionally, bulb P decreased from 2.06 in control to 1.60 mg g^−^¹ at 60-minute exposure, and root K decreased from 10.91to 7.89 mg g^−^¹ at 60-minute exposure.

### 3.3 Effect of He-Ne laser and UV_A+B_ on nutrient use efficiency

Two-way ANOVA demonstrated that radiation type significantly affected nutrient efficiency across all measured parameters-uptake efficiency, use efficiency, and productive efficiency for N, P, and K. While radiation dose significantly affected N uptake efficiency and use efficiency. Notably, the radiation × dose interaction was significant for all nutrient efficiency parameters, indicating that He-Ne laser and UV_A+B_ radiation elicit fundamentally different efficiency responses at varying doses (**Supplementary Table 1**).

Clearly, He-Ne laser treatments resulted in significantly higher nutrient efficiency values compared to UV_A+B_ and control treatments. He-Ne laser treatment produced the highest uptake efficiency (75.42, 18.12, and 82.43%), use efficiency (42.48, 9.50, and 38.98%), and productive efficiency (56.72, 110.28 and 66.17 kg/kg) for N, P, and K, respectively. Also, UV _A+B_ treatment improved N, P, and K uptake efficiency (46.33%, 12.05 and 58.79%) and N use efficiency (23.79%) compared to untreated plants. While UV_A+B_ treatment reduced use efficiency of P and K (5.80 and 25.67%, respectively) and productive efficiency for N, P, and K (46.79, 90.98, and 54.59 kg/kg, respectively). However, these decreases did not reach statistical significance compared to control plants (**Supplementary Table 1**).

**Figure 1:**
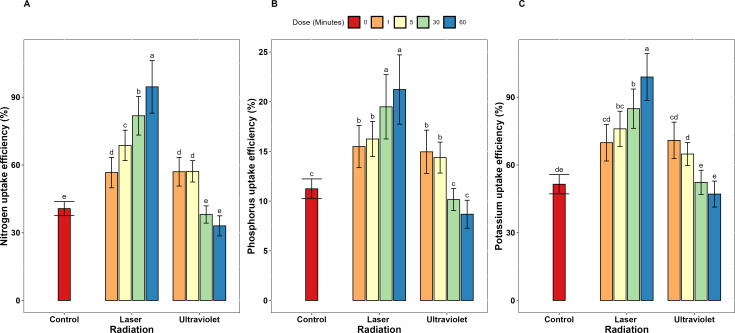
Nutrient uptake efficiency for **(A)** N, **(B)** P, and **(C)** K in garlic irradiated with different exposure doses (0, 1, 5, 30, and 60) of He-Ne laser and ultraviolet radiation (UV_A+B_). Error bars represent standard deviation (SD). Different letters above bars indicate significant differences at P < 0.05 (Tukey’s HSD test).

**Figure 2:**
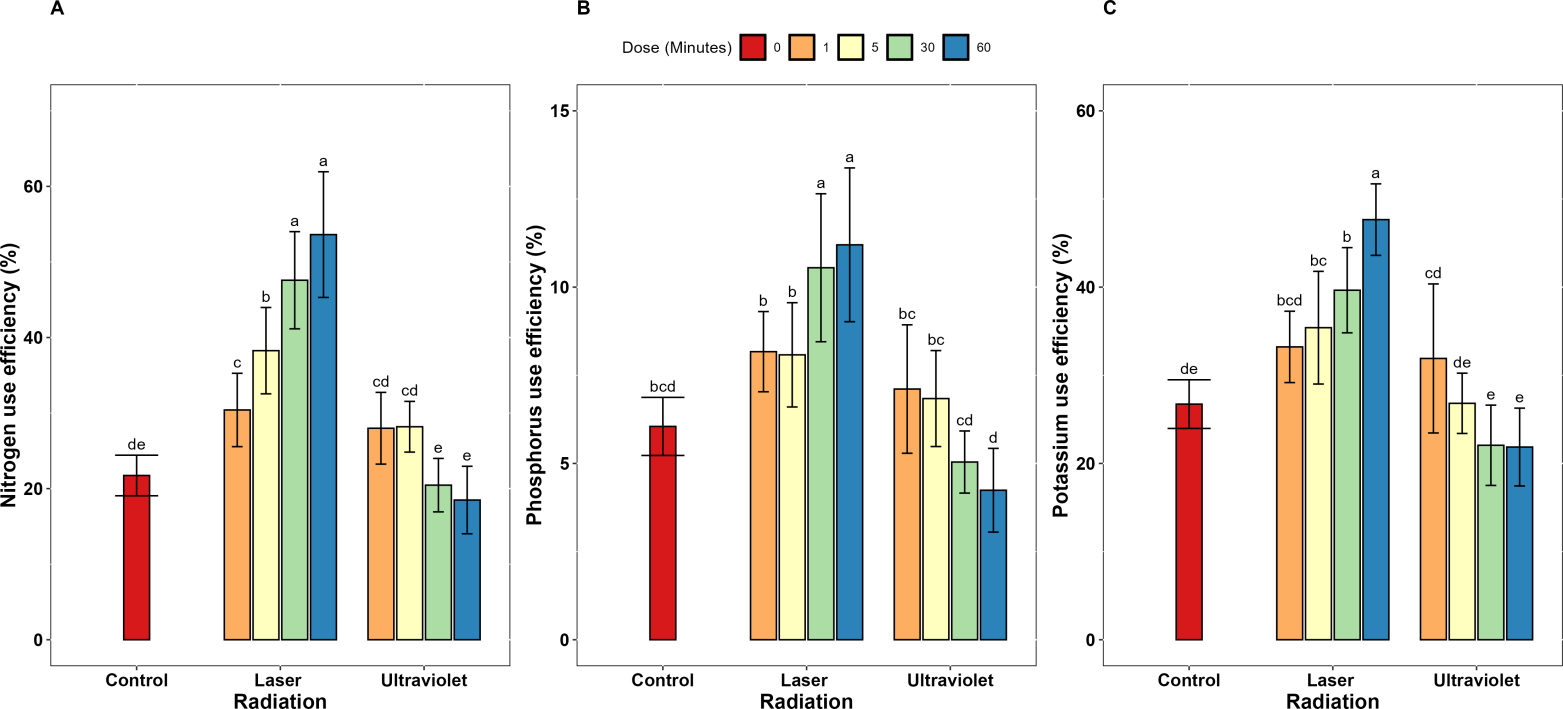
Nutrient use efficiency for **(A)** N, **(B)** P, and **(C)** K in garlic irradiated with different exposure doses (0, 1, 5, 30, and 60) of He-Ne laser and ultraviolet radiation (UV_A+B_). Error bars represent standard deviation (SD). Different letters above bars indicate significant differences at P < 0.05 (Tukey’s HSD test).

**Figure 3:**
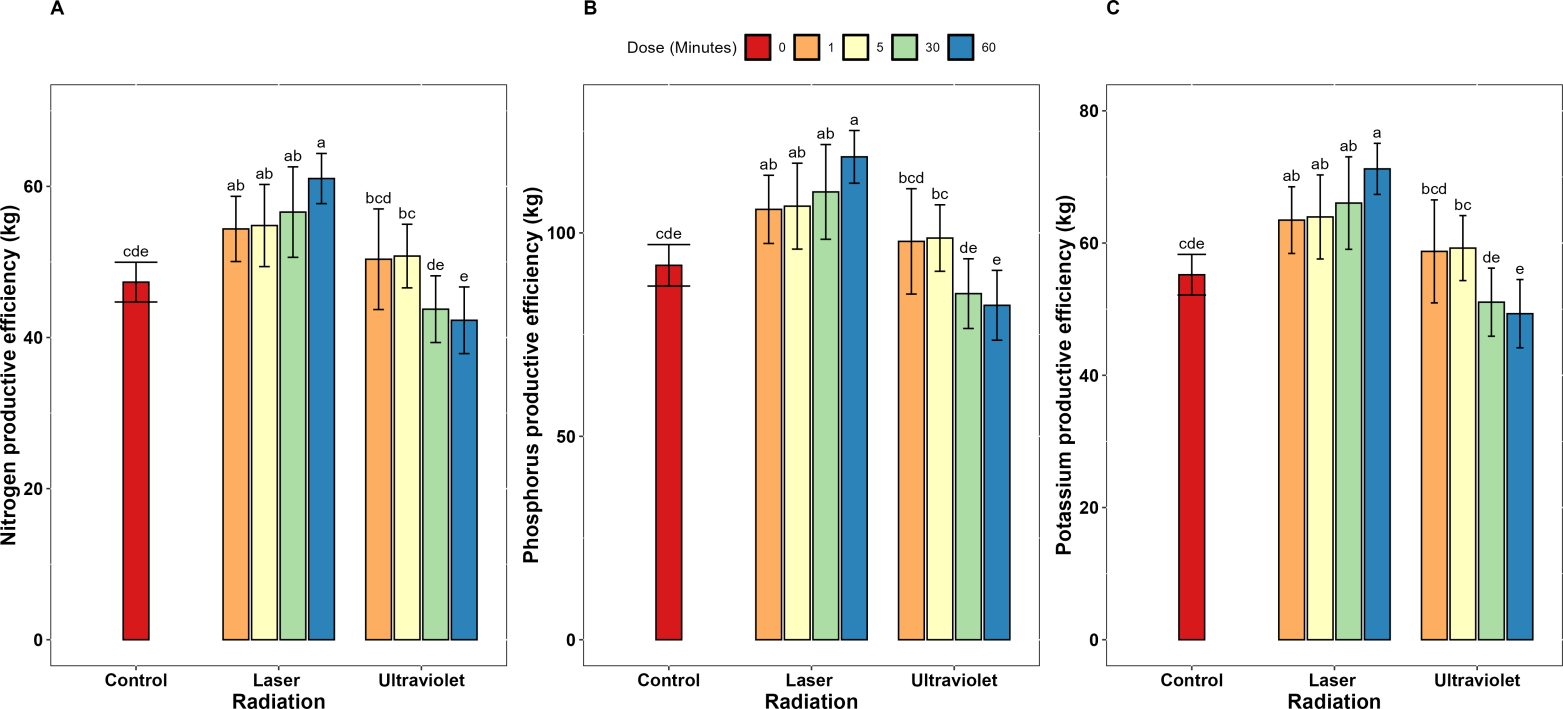
Nutrient productive efficiency for **(A)** N, **(B)** P, and **(C)** K in garlic irradiated with different exposure doses (0, 1, 5, 30, and 60) of He-Ne laser and ultraviolet radiation (UV_A+B_). Error bars represent standard deviation (SD). Different letters above bars indicate significant differences at P < 0.05 (Tukey’s HSD test).

Concerning the dose-dependent effects, He-Ne laser exposure duration demonstrated a clear dose-dependent relationship with nutrient use efficiency across all measured parameters. Extending exposure from 1 to 60 minutes resulted in progressive improvements in N, P, and K uptake efficiency increased (67.1%, 37.1%, and 41.6% respectively), use efficiency (76.3%, 37.1%, and 43.5% respectively). Also, productive efficiency increased by 12.2% for all three elements. At 60-minute exposure, He-Ne laser achieved maximum N, P and K uptake efficiency values of 94.59, 21.24 and 98.95% (**Figures 1A–C**). Also, N, P, and K use efficiency similarly peaked at 60-minute exposure with 53.63% 11.20 and 47.68% (**Figures 2A–C**). Moreover, N, P and K productive efficiency also reached maximum values of 61.05, 118.7, and 71.22 kg/kg (**Figures 3A–C**).

Unlike He-Ne laser treatment, extending UV_A+B_ exposure from 1 to 60 minutes resulted in significant reductions in nutrient efficiency parameters across all measures. Specifically, substantial reduction was recorded in uptake efficiency (42.0, 41.9 and 33.5%) and use efficiency (33.9, 40.2 and 31.5%) of N, P and K, respectively. Data presented in **Supplementary Table 1** clearly shows that UV_A+B_ radiation exhibited a biphasic dose-response pattern across all nutrient efficiency parameters. At low exposure durations (1 and 5 minutes), UV_A+B_ treatment enhanced nutrient uptake efficiency compared to control: N uptake efficiency increased by 40.1 and 40.6%, P uptake efficiency by 33.3 and 28.1%, and K uptake efficiency by 37.7 and 26.0% respectively. Similarly, nutrient use efficiency improved at low doses: N use efficiency increased by 28.7 and 29.7%, P use efficiency by 17.2 and 12.9%, and K use efficiency by 19.3 and 0.3% respectively. Productive efficiency also benefited from short exposures, increasing by 6.4% and 7.3% for each element.

In stark contrast, extended UV_A+B_ exposures (30 and 60 minutes) negatively declined all nutrient efficiency parameters. N uptake efficiency decreased by 6.5 and 18.8%, P uptake efficiency by 9.8 and 22.6%, and K uptake efficiency by 1.6 and 8.5% respectively. Nutrient use efficiency similarly declined at extended doses: N use efficiency decreased by 5.9 and 14.9%, P use efficiency by 16.6 and 29.9%, and K use efficiency by 17.5 and 18.2% respectively. Productive efficiency also decreased at extended exposures, declining by 7.5% and 10.7% for all three elements.

### 3.4 Principal component analysis

**Figure 4:**
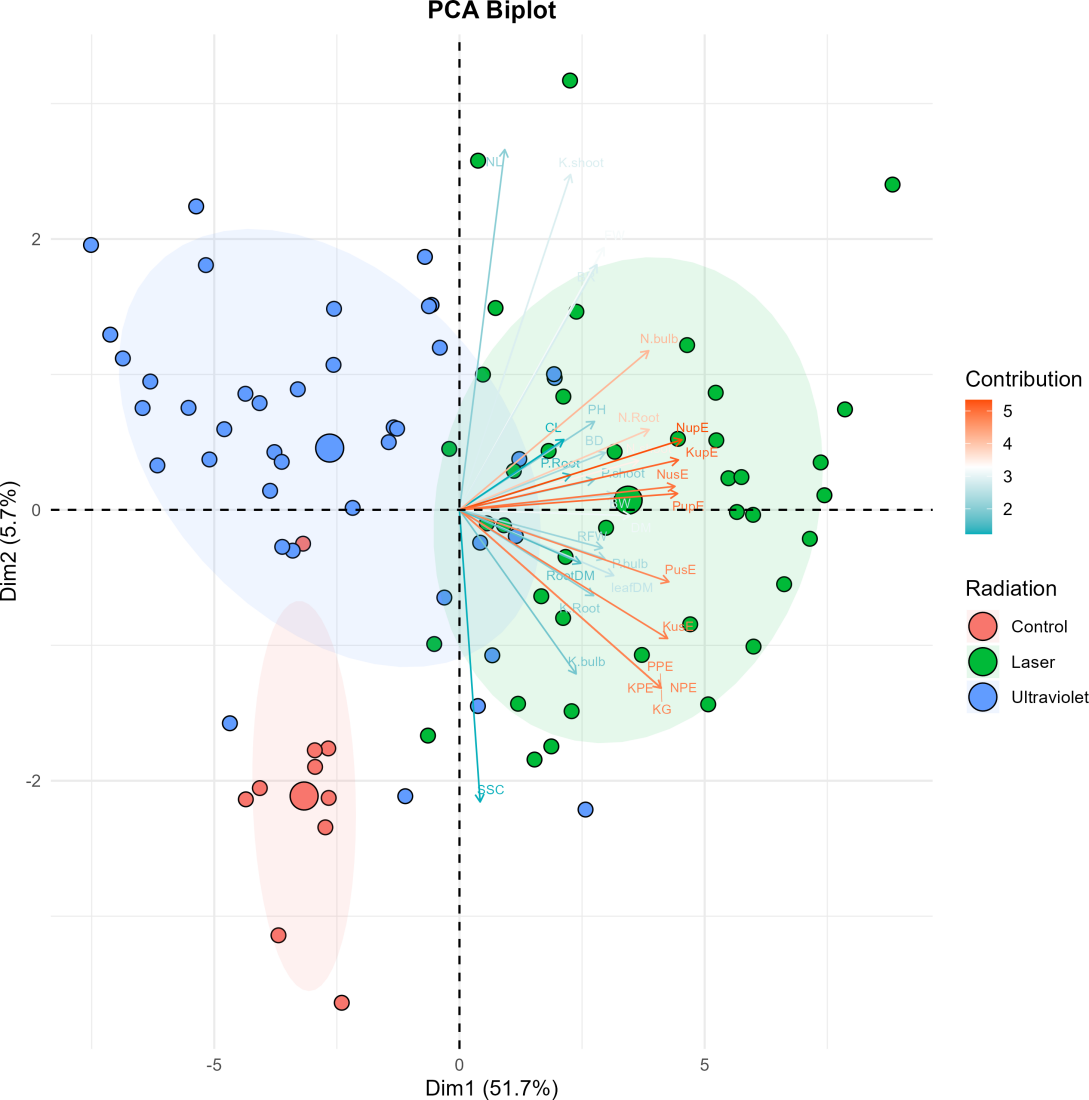
PCA biplot of growth, yield, and mineral content and nutrient efficiency of garlic treated with different He-Ne and UV_A+B_ radiation (0, 1, 5, 30 and 60 min). For an abbreviation description see the list of abbreviations. PH, plant height; FW, shoot fresh weight; BW, bulb fresh weight; DM, bulb dry matter; SSC, soluble solids content; CL, chlorophyll content; BD, bulb diameter; NL, number of leaves; BR, bulbing ratio; KG, bulb yield; N.shoot, nitrogen content in shoot; P.shoot, phosphorus content in shoot; K.shoot, potassium content in shoot; N.bulb, nitrogen content in bulb; P.bulb, phosphorus content in bulb; K.bulb, potassium content in bulb; N.Root, nitrogen content in root; P.Root, phosphorus content in root; K.Root, potassium content in root; PPE, phosphorus productive efficiency; KPE, potassium productive efficiency; NupE, nutrient uptake efficiency; PupE, phosphorus uptake efficiency; KupE, potassium uptake efficiency; NusE, nutrient use efficiency; PusE, phosphorus use efficiency; KusE, potassium use efficiency; RFW, root fresh weight; leafDM, leaf dry matter; RootDM, root dry matter.

The effects of He-Ne laser and UVA+B radiation on garlic growth, yield, mineral content, and nutrient efficiency were illustrated with the help of elliptical PCAs (**Figure 4**). The closed and linear eigenvector demonstrated a noteworthy association between traits at discriminating levels of radiation treatment. The explained variation for growth and nutrient traits were PC1 51.7% and PC2 5.7% with a cumulative of 57.4%. The nutrient uptake efficiency and nutrient use efficiency traits for N, P and K were closely clustered toward the PC1 by closely associating nutrient accumulation in bulbs and biomass traits (bulb dry matter, bulb weight, plant height).

The PCA biplot revealed that He-Ne laser treatments exhibited markedly positive PC1 scores (1 min: 0.85; 5 min: 1.97; 30 min: 4.33; 60 min: 6.61) showing a dose-dependent response, while control plants showed negative PC1 scores (-3.17) and UV A+B treatments clustered in the negative PC1 region (1–60 min: -0.18 to -5.79). The PCA analysis demonstrated that He-Ne laser pre-treatment induced a comprehensive, dose-dependent multivariate phenotype distinct from both control and UV A+B treated plants.

### 3.5 Polynomial response surface model

The second-order polynomial RSM model was highly significant (F(5, 84) = 27.0, p < 0.001), explaining 59.4% of the total variation in bulb yield (Adjusted R² = 0.5936). Compared to the non-irradiated control, both radiation types produced significant initial increases in yield, He-Ne laser by 1,303.87 kg/feddan (p < 0.001) and UV_A+B_ by 669.27 kg/feddan (p = 0.046), however, their dose-response trajectories were markedly divergent.

**Figure 5:**
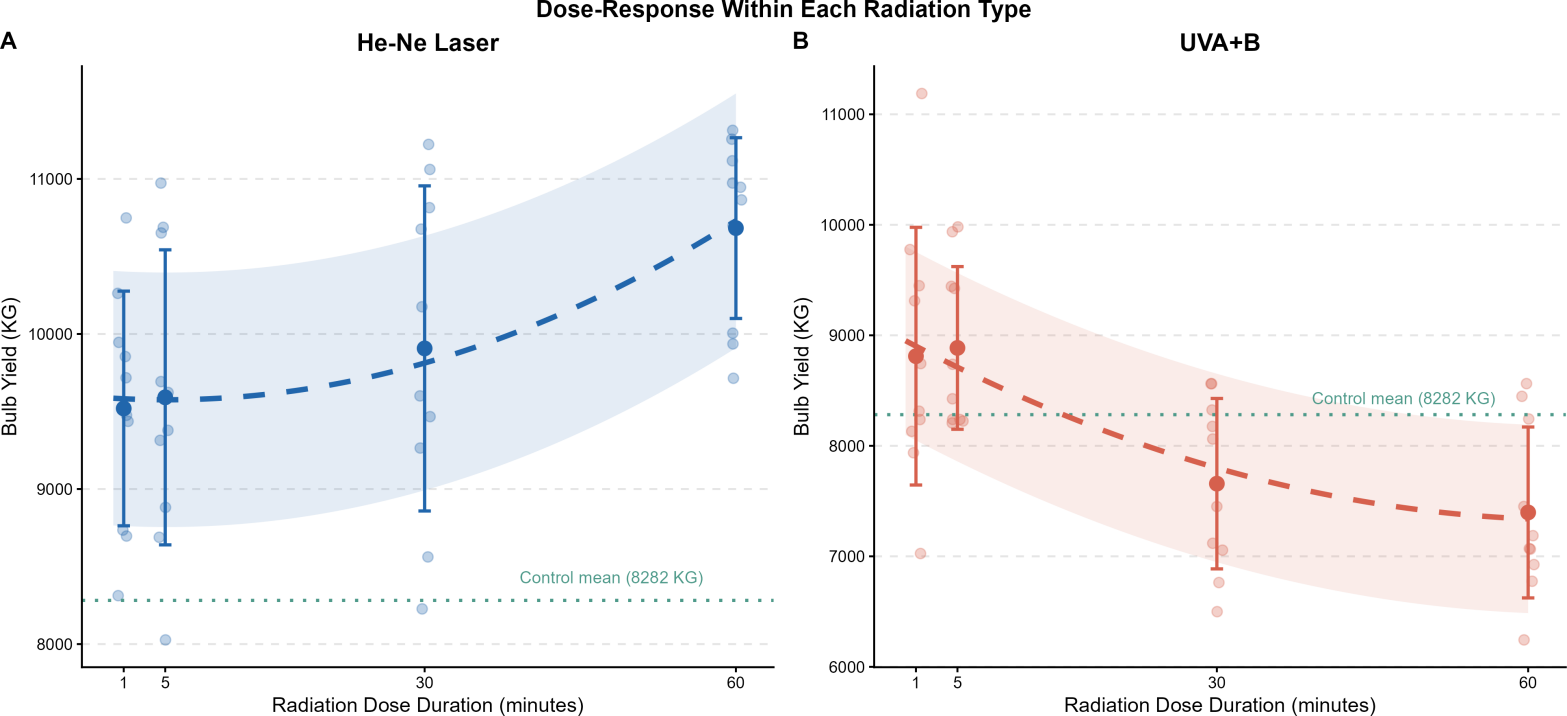
Dose-response relationship between radiation exposure duration **(minutes)** and garlic bulb yield (kg fed^−^¹) within each radiation type. Panel **(A)** He-Ne Laser; Panel **(B)** UV_A+B_ radiation. Filled circles represent group means ± SD; the dashed line indicates the RSM-fitted second-order polynomial curve; the shaded band represents ± one residual standard deviation around the fitted curve; and the horizontal dotted green line denotes the mean yield of the non-irradiated control group (8,281.88 kg fed^−^¹) as a reference baseline.

For the He-Ne laser treatment, a strong and highly significant positive interaction between radiation type and dose duration was identified (p < 0.001), with each additional minute of exposure contributing an estimated 45.9 kg/feddan increase in yield. Mean yield increased progressively from 9,519.62 kg/feddan at 1 minute to a maximum of 10,682.88 kg/feddan at 60 minutes (**Figure 5A**). The model predicted the optimal dose at the upper experimental boundary of 60 minutes, yielding a maximum predicted value of 10,730.22 kg/feddan.

In stark contrast, UV_A+B_ radiation exhibited a negative dose-response relationship. Although mean yields at 1 and 5 minutes (8,811.25 and 8,885.88 kg/feddan, respectively) marginally exceeded the control baseline, prolonged exposure was clearly detrimental, with yields declining to 7,657.50 kg/feddan at 30 minutes and 7,397.00 kg/feddan at 60 minutes, both well below the control mean of 8,281.88 kg/feddan (**Figure 5B**).

## 4 Discussion

Several investigations have demonstrated the positive effects of photostimulatory strategies such as He-Ne laser and UV radiation on seed priming (Farooq et al., 2019; Escobar-Hernández et al., 2024), improving growth and yield traits (Osman et al., 2009; Liu et al., 2013) as well as enhancing plant tolerance against abiotic stresses (Aslam et al., 2022; Escobar-Hernández et al., 2025). This study comparatively assessed the photostimulatory effects of He-Ne laser and UV_A+B_ radiation on garlic growth, yield, mineral content, and nutrient use efficiency to elucidate the mechanisms underlying these radiation treatments.

Our study clearly reported that He-Ne laser treatment produced progressive, dose-dependent improvements across all measured parameters. At 60-minute exposure to He-Ne laser, significant improvements were recorded in plant height, shoot fresh weight, total yield, bulb fresh weight, bulb dry matter, and bulbing ratio. These enhancements are consistent with findings across diverse plant species. Whereas, He-Ne laser enhances shoot and root biomass, leaf area, and leaf number in sunflower, wheat, ashwagandha, *Moringa oleifera* and *Lavatera thuringiaca* (Shafique et al., 2017; Mahmood et al., 2021; Thorat et al., 2021; Aslam et al., 2022; Budzeń et al., 2023), and increases yield in fennel, coriander and soybean (Osman et al., 2009; Klimek-Kopyra et al., 2022). The consistency of these effects across these diverse species might suggest a fundamental physiological mechanism shared across plant species.

In addition to growth improvements, significant increases in chlorophyll content were recorded in response to He-Ne laser for 60 minutes, indicating enhanced photosynthetic apparatus development. However, direct measurement of photosynthetic rate is required to confirm enhanced photosynthetic capacity. The increased chlorophyll content likely provides the metabolic foundation for improved biomass accumulation by increasing light capture and photosynthetic energy availability. In this regard, He-Ne laser might activate phytochrome-mediated metabolic pathways through resonant interaction with the Pr form of phytochrome (Dudareva, 2024), which triggers downstream signaling cascades that enhance photosynthetic enzyme activity and nutrient transporter expression (Qiu et al., 2017; Aslam et al., 2022). This phytochrome-mediated mechanism might explain the consistency of laser effects across diverse plant species, as phytochrome functions as a universal photoreceptor in plants.

A novel finding of current study is that He-Ne laser treatment dramatically improved mineral content of N, P and K and their efficiency across all plant tissues. Shoot N, P, and K increased by 26.3, 13.6, and 23.0% respectively, while bulb tissues showed more pronounced increases (N 50.0, P 13.1, K 8.9%), and root tissues recorded the most substantial improvements (N 55.0, P 17.0, K 9.1%) at 60-minute exposure. These tissue-specific nutrient patterns reflect differential allocation between source and sink tissues: root tissues showed the highest nutrient increases because they function as primary uptake organs directly exposed to soil nutrients; bulb tissues accumulated substantial nutrient quantities supporting storage function and consumer nutritional value; shoot tissues showed intermediate increases, consistent with their role in photosynthetic energy production and nutrient translocation to storage organs. This differential tissue response indicates that He-Ne laser might enhance nutrient transporter expression and activity in roots, facilitating enhanced nutrient uptake from soil, followed by preferential translocation and accumulation in economically important bulb tissues (Różanowski et al., 2020; Banisharif and Amooaghaie, 2025).

Correspondingly, all nutrient efficiency parameters increased substantially: N productive efficiency (+29.0%), N uptake efficiency (+132.5%), and N use efficiency (+146.6%), accompanied by similar increases in P and K efficiency. The exceptional magnitude of these improvements suggests that He-Ne laser treatment fundamentally enhances the plant’s capacity to acquire and utilize soil nutrients. This likely reflects laser-induced upregulation of nutrient transporter genes, enhanced photosynthetic energy availability for active nutrient uptake, and improved nutrient partitioning to economically important tissues (Qiu et al., 2017; Hassan et al., 2024). These results align with previous research demonstrating that He-Ne laser enhances mineral content and nutrient uptake and utilization efficiency across several plant species (Shafique et al., 2017; Okla et al., 2022; Amooaghaie and Banisharif, 2025). Specifically, He-Ne laser enhances uptake of essential minerals (N, P, Mg, Fe, Zn, and Cu) while decreasing cadmium accumulation through down-regulation of cadmium transport genes and activating protective antioxidant defense systems, which improves overall mineral nutrient status (Gao et al., 2016). Collectively, these findings might indicate that He-Ne laser activates fundamental metabolic pathways for comprehensive nutrient accumulation across multiple plant tissues, with important implications for enhancing both plant defense mechanisms and human nutritional intake.

In contrast to the consistent and positive He-Ne laser effects, UV_A+B_ radiation showed a biphasic dose-response pattern, reflecting the dual role of UV as both eustress (beneficial stress) at low doses and distress (harmful stress) at high doses. In this study, short UV_A+B_ exposure (1–5 minutes) produced a coordinated eustress response that simultaneously enhanced growth, mineral accumulation, and nutrient efficiency across all measured parameters. Practically, at 5-minute exposure, the maximum benefits were recorded in plant height increased, shoot fresh weight, bulb fresh weight, root fresh weight, chlorophyll content, bulb diameter, bulbing ratio, and total yield. Mineral content also increased substantially; shoot N, P, and K increased by 18.7, 13.2, and 20.0% respectively, with similar increases in bulb tissues (N + 20.1%, P + 3.9%) and root tissues (N + 7.2%). Correspondingly, N, P, and K use efficiency increased by 29.7, 12.9, and 0.3% respectively, while productive efficiency increased by 7.3% for each element.

This enhancement might reflect activation of plant defense mechanisms through increased production of secondary metabolites including flavonoids, phenolics, and antioxidant enzymes (Mmbando, 2024; Li et al., 2024), which in turns improve growth and productivity and plant tolerance to environmental stresses. These findings are in agreement with previous studies demonstrating that controlled UV supplementation improves biomass and antioxidant content in leafy greens such as kale, sweet basil, and lettuce, as well as induces water stress tolerance in tomato and bell pepper, as well as improves growth and nutritional properties of lettuce grown in artificial light plant factories (Kang et al., 2018; Chen et al., 2019; Lee et al., 2019; He et al., 2021; Yan et al., 2021; Kang et al., 2022; Escobar-Hernández et al., 2024). The simultaneous improvements in all measured parameters indicate that low-dose UV_A+B_ radiation might activate a metabolic process that enhances plant productivity and nutritional quality within an optimal dose window of approximately 5 minutes for garlic.

Conversely, extended UV_A+B_ exposure (30–60 minutes) exceeded the plant’s capacity to maintain protective mechanisms, resulting in a coordinated distress response that negatively affect growth, mineral content, and nutrient efficiency across all measured parameters. At 60-minute exposure substantial decreases were recorded in shoot dry matter (−19.7%), bulb fresh weight (−7.2%), bulb dry matter (−0.6%), bulb diameter (−6.8%), and total bulb yield (−10.7%). NPK content also declined across all tissues: bulb (−5.6%, −22.3%, −8.6%), and root (−11.5%, −8.5%, −27.7%), and N and P content in shoot (−15.3 and −6.0%, respectively). Also, nutrient efficiency parameters correspondingly declined: N productive efficiency (−10.7%), N uptake efficiency (−18.8%), and N use efficiency (−14.9%) compared to control.

This decline may reflect photosystem I and II damage, reduced photosynthetic efficiency, and increased oxidative stress that collectively impair nutrient acquisition and utilization. This negative response aligns with observations in rice, lettuce, radish and wheat, where UV supplementation reduced leaf area, biomass, chlorophyll content, and photochemical efficiency (Zhang et al., 2014; Abu-Elsaoud and Hassan, 2015; Li et al., 2024). Taken together, these findings suggest that the eustress-to-distress transition occurs across multiple plant species, with the threshold for garlic occurring at approximately 5 minutes, beyond which accumulated photochemical damage and oxidative stress outweigh protective benefits.

The results of current study clearly show that the fundamental difference between He-Ne laser and UV_A+B_ radiation lies in their dose-response relationships and underlying mechanisms. He-Ne laser produces consistent and progressive improvements across all doses without evidence of photochemical damage or growth inhibition, suggesting activation of a sustainable metabolic pathway. While UV_A+B_ radiation exhibits a narrow dose window (1–5 minutes) beyond which protective mechanisms are overwhelmed by photochemical damage. This mechanistic difference reflects distinct modes of action: He-Ne laser activates phytochrome-mediated signaling pathways that enhance nutrient acquisition and photosynthetic capacity in a dose-dependent manner (Swathy et al., 2023; Dudareva, 2024; Amooaghaie and Banisharif, 2025), while UV_A+B_ radiation induces eustress at low doses through secondary metabolite accumulation but exceeds the plant’s capacity to maintain protective mechanisms at high doses, resulting in oxidative stress and metabolic dysfunction (Hollósy, 2002).

PCA confirmed these mechanistic differences by integrating all measured traits into a multivariate framework (**Figure 4**). PC1 revealed that He-Ne laser treatments induced a coordinated nutrient-efficient pattern, with nutrient uptake and use efficiency traits strongly associated with nutrient accumulation in storage organs and biomass. Laser PC1 scores increased dose-dependently (0.85 to 6.61), demonstrating progressive enhancement of nutrient acquisition. In contrast, UV_A+B_ treatments clustered in the negative PC1 region (−0.18 to −5.79) with high variance, indicating that UV_A+B_ does not induce a consistent nutrient-efficient pattern. This multivariate segregation might improve that He-Ne laser activates an integrated nutrient-efficient pattern through phytochrome-mediated signaling, while UV_A+B_ radiation suppresses this pattern, particularly at extended exposure.

The most notable outcome of the RSM analysis was the strong, positive, and dose-dependent effect of He-Ne laser irradiation on bulb yield. The highly significant interaction between laser treatment and dose duration (β = 45.95, *p* < 0.001) indicates that the biostimulatory effect of the laser was not merely additive but intensified with increasing exposure time, with each additional minute contributing an estimated 45.9 kg/feddan to the final yield. Absence of a significant quadratic term (p = 0.141) suggests no detectable curvature within the tested 0–60 minutes range; however, confidence in this conclusion is limited, as non-significant quadratic terms may also indicate low statistical power, model misspecification, or insufficient curvature detectable. Therefore, the true optimal dose may extend beyond the upper boundary tested. The model-predicted maximum yield of 10,730.22 kg/feddan at 60 minutes, representing a 29.6% improvement over the control, underscores the practical agronomic potential of He-Ne laser pre-sowing treatment for enhancing garlic productivity. This pronounced dose-response for garlic highlights the species-specific nature of He-Ne laser biostimulation, which depends on laser type, wavelength, intensity, and exposure duration. In contrast to our results, previous research reported that the low exposure time, 2 minutes, was more effective than long exposure time in enhancing plant tolerance against abiotic stresses (Mahmood et al., 2021; Aslam et al., 2022). However, this striking 30-fold difference in required exposure time between garlic and wheat/sunflower (60 versus 2 minutes) can be explained by tissue size: because He-Ne laser light penetrates only 2–3 mm into plant tissue, large garlic cloves require extended exposure to reach and stimulate cells throughout the entire structure, whereas small seeds need minimal exposure since light penetrates the entire seed (Możdżeń et al., 2020; Amooaghaie and Banisharif, 2025). This tissue penetration limitation accounts for the species-specific exposure requirements and suggests that optimizing laser parameters for each crop requires consideration of tissue size and structure.

Conversely, the RSM analysis revealed that UV_A+B_ radiation exerted a predominantly inhibitory effect on bulb yield with increasing dose. Although the initial linear coefficient was marginally significant (*p* = 0.046), the overall response surface declined with dose, and the model identified 0 minutes as the optimal UV_A+B_ exposure. The observed mean yields at 30 and 60 minutes fell well below the control baseline, demonstrating that prolonged UV_A+B_ irradiation is detrimental rather than beneficial under the conditions of this experiment (Nawkar et al., 2013; Kataria et al., 2014). These contrasting response surfaces highlight the critical importance of radiation type and dose selection in pre-sowing seed treatment protocols.

Based on this study, He-Ne laser pre-treatment represents a novel technology-driven approach to optimize nutrient use efficiency in garlic cultivation, directly addressing sustainable fertilization strategies that move beyond short-term yield maximization. The 29.0% yield increase combined with exceptional nutrient efficiency improvements (N uptake efficiency +132.5%, N use efficiency +146.6%) demonstrates that He-Ne laser treatment enhances both productivity and quality while reducing chemical fertilizer dependency. By improving nutrient acquisition capacity, thereby laser pre-treatment decreases production costs and environmental impacts associated with excessive nutrient application. The species-specific optimization of laser exposure (60 minutes for garlic) warrants investigation across other economically important crops to develop standardized protocols for precision fertilization. Integration with existing sustainable practices, including organic amendments and biofertilizers, offers a comprehensive pathway to maintain productivity while reducing chemical input dependency and supporting long-term environmental sustainability. However, practical application faces several obstacles: (1) high capital costs may limit accessibility for small-scale farmers; (2) standardized protocols for different crops and conditions are not yet established; (3) energy requirements may offset environmental benefits in regions with high-carbon electricity; (4) farmer awareness and training are necessary for widespread adoption.

### 4.1 Future research directions

Future research might focus on two key areas to advance He-Ne laser technology for garlic cultivation. First, cultivar-specific optimization should be investigated to determine whether the optimal 60-minute dose applies uniformly across different garlic varieties or requires cultivar-dependent adjustments. Second, molecular mechanisms underlying He-Ne laser-induced nutrient bioconcentration should be elucidated through transcriptomic and proteomic analysis to identify key genes and pathways involved in enhanced nutrient uptake and utilization efficiency. These research directions might facilitate the development of evidence-based protocols for sustainable garlic production and potential extension to other vegetable crops.

## 5 Conclusions

This study demonstrated that He-Ne laser treatment produces progressive, dose-dependent improvements across all measured parameters. The 60-minute He-Ne laser treatment produced significant improvements in plant height (20.5%), total yield (29.0%), chlorophyll content (35.1%), and nutrient use efficiency, with N uptake efficiency and N use efficiency enhanced by 132.5% and 146.6%, respectively. In contrast, UV_A+B_ radiation exhibited a biphasic dose-response, with low doses (1–5 minutes) showing moderate benefits and high doses (60 minutes) inducing stress that reduced shoot dry matter (−19.7%), fresh bulb weight (−7.2%), bulb yield (−10.7%), and N, P, and K use efficiency (−14.9%, −29.9%, and −18.2%, respectively). Taken together, these findings highlight He-Ne laser technology as a sustainable strategy to improve crop yields and nutritional quality in modern agriculture. Future research should focus on elucidating the molecular mechanisms of nutrient bioconcentration enhancement and optimizing laser parameters for different garlic cultivars and field conditions.

## Data Availability

The original contributions presented in the study are included in the article/**Supplementary Material**. Further inquiries can be directed to the corresponding author.

## Supplementary material

Supplementary Table 1 Nutrient uptake efficiency, nutrient use efficiency, and nutrient productive efficiency of garlic in response to various doses of He-Ne laser and UV_A+B_ radiation.
