# Supplementary material for: Helium-neon laser pre-treatment optimizes nutrient use efficiency and yield in garlic plant
Source: Front Plant Sci. 2026 May 21;17:1821225. doi: 10.3389/fpls.2026.1821225 (PMC13233445; doi:10.3389/fpls.2026.1821225)
Supplement: Supplementary Table 1 — Nutrient uptake efficiency, nutrient use efficiency, and nutrient productive efficiency of garlic in response to various doses of He-Ne laser and UVA+B radiation. [file Table1.docx]

Supplementary Material

# Supplementary Data

Does not apply.

# Supplementary Figures and Tables

## Supplementary Figures

Does not apply.

## Supplementary Tables

**Supplementary Table 1.** Nutrient uptake efficiency, nutrient use efficiency, and nutrient productive efficiency of garlic in response to various doses of He-Ne laser and UV_A+B_ radiation.

| **Radiation**  **/Dose (min.)** | | **Nutrient efficiency** | | | | | | | | | | | | | | | | |
| --- | --- | --- | --- | --- | --- | --- | --- | --- | --- | --- | --- | --- | --- | --- | --- | --- | --- | --- |
|  |  | Nutrient uptake efficiency(NUE),  (%) | | | | | Nutrient use efficiently (NUtE),  (%) | | | | | | Nutrient productive efficiency (NPE),  (kg/kg) | | | | | |
|  |  | **N** | **P** | | **K** | | **N** | | **P** | | **K** | | **N** | | **P** | | **K** | |
| **Control** | -- | 40.69±3.13e | 11.23±0.99d | | 51.48±4.26d | | 21.75±2.69de | | 6.05±0.82bcd | | 26.73±2.73de | | 47.33±2.63cde | | 92.02±5.11cde | | 55.21±3.07cde | |
| **UV_A+B_** | 1 | 57.01±6.31d | 14.97±2.18c | | 70.89±8.07c | | 27.98±4.74cd | | 7.09±1.83bc | | 31.90±8.45cd | | 50.35±6.66bcd | | 97.9±12.95bcd | | 58.74±7.77bcd | |
|  | 5 | 57.20±4.73d | 14.39±1.55c | | 64.85±5.12c | | 28.20±3.37cd | | 6.83±1.36bc | | 26.82±3.41de | | 50.78±4.21bcd | | 98.73±8.18bcd | | 59.24±4.91bcd | |
|  | 30 | 38.06±3.81e | 10.14±1.11d | | 52.31±5.36d | | 20.47±3.53e | | 5.05±0.88cd | | 22.07±4.56e | | 43.76±4.41de | | 85.08±8.57de | | 51.05±5.14de | |
|  | 60 | 33.04±4.43e | 8.69±1.42d | | 47.12±5.74d | | 18.50±4.47e | | 4.24±1.19d | | 21.87±4.41e | | 42.27±4.42e | | 82.19±8.59e | | 49.31±5.16e | |
|  | **Mean** | **46.33±12.02** | **12.05±3.13** | | **58.79±11.31** | | **23.79±5.9** | | **5.8±1.78** | | **25.67±6.77** | | **46.79±6.18** | | **90.98±12.02** | | **54.59±7.21** | |
| **He-Ne laser** | 1 | 56.62±6.73d | 15.49±2.13c | | 69.86±8.1c | | 30.42±4.85c | | 8.17±1.14b | | 33.23±4.05bcd | | 54.40±4.32ab | | 105.77±8.41ab | | 63.46±5.04ab | |
|  | 5 | 68.66±6.72c | 16.26±1.78bc | | 75.99±7.8bc | | 38.27±5.72b | | 8.08±1.47b | | 35.38±6.4bc | | 54.81±5.44ab | | 106.57±10.57ab | | 63.94±6.34ab | |
|  | 30 | 81.79±8.56b | 19.49±3.23ab | | 84.90±8.72b | | 47.59±6.44a | | 10.55±2.09a | | 39.65±4.84b | | 56.61±5.99ab | | 110.08±11.65ab | | 66.05±6.99ab | |
|  | 60 | 94.59±11.65a | 21.24±3.5a | | 98.95±10.33a | | 53.63±8.3a | | 11.20±2.16a | | 47.68±4.06a | | 61.05±3.33a | | 118.70±6.48a | | 71.22±3.89a | |
|  | **Mean** | **75.42±16.62** | **18.12±3.56** | | **82.43±13.93** | | **42.48±10.9** | | **9.5±2.21** | | **38.98±7.33** | | **56.72±5.39** | | **110.28±10.49** | | **66.17±6.29** | |
| **Analysis of variance (ANOVA)** | | | | | | | | | | | | | | | | | | |
| F_(Radiations)_ | | 227.96*** | | 93.66*** | | 135.26*** | | 152.61*** | | 64.67*** | | 75.51*** | | 47.50*** | | 47.51*** | | 47.51*** |
| F_(Dose)_ | | 4.48** | | 0.24 | | 1.25 | | 6.22*** | | 0.19 | | 2.56 | | 1.16 | | 1.16 | | 1.16 |
| F_(Radiation* Dose)_ | | 90.56*** | | 36.35*** | | 51.23*** | | 42.47*** | | 19.48*** | | 22.42*** | | 11.53*** | | 11.53*** | | 11.54*** |

Data are presented as mean ± SD (n=15). Values represent measurements from 15 plants (5 per replicate plot) in a randomized complete block design with three replicates. SD reflects variation among all sampled plants. Means followed by the same letter are not significantly different according to Tukey's multiple range test. Significance levels: * p < 0.05, ** p < 0.01, *** p < 0.001.
